# Gene-edited vero cells as rotavirus vaccine substrates

**DOI:** 10.1016/j.jvacx.2019.100045

**Published:** 2019-10-08

**Authors:** Nichole Orr-Burks, Jackelyn Murray, Weilin Wu, Carl D. Kirkwood, Kyle V. Todd, Les Jones, Abhijeet Bakre, Houping Wang, Baoming Jiang, Ralph A. Tripp

**Affiliations:** a Department of Infectious Diseases, College of Veterinary Medicine, University of Georgia, Athens, GA, USA; b Enteric and Diarrheal Diseases, Bill & Melinda Gates Foundation, Seattle, WA, USA; c Division of Viral Diseases, National Center for Immunization and Respiratory Diseases, Centers for Disease Control and Prevention, Atlanta, GA, USA

**Keywords:** Rotavirus, Vaccine substrates, CRISPR-Cas9, Enhanced vaccine cell line

## Abstract

**Background:**

Rotavirus (RV) is a leading cause of severe gastroenteritis globally and can cause substantial morbidity associated with gastroenteritis in children <5 years of age. Orally administered live-attenuated RV vaccines offer protection against disease but vaccination efforts have been hampered by high manufacturing costs and the need to maintain a cold chain.

**Methods:**

A subset of Vero cell host genes was identified by siRNA that when knocked down increased RV replication and these anti-viral host genes were individually deleted using CRISPR-Cas9.

**Results:**

Fully-sequenced gene knockout Vero cell substrates were assessed for increased RV replication and RV vaccine antigen expression compared to wild type Vero cells. The results showed that RV replication and antigen production were logs higher in Vero cells having an *EMX2* gene deletion compared to other Vero cell substrates tested.

**Conclusions:**

We used siRNAs to screen for host genes that negatively affected RV replication, then CRISPR-Cas9 gene editing to delete select genes. The gene editing led to the development of enhanced RV vaccine substrates supporting a potential path forward for improving RV vaccine production.

## 1 Introduction

Rotavirus (RV) causes diarrhea-associated hospitalization in infants <5 years of age in every country globally, and 125,000–200,000 deaths occur each year in predominantly in less developed settings in Africa and Asia [5], [6]. RVs are non-enveloped, icosahedral viruses that belong to the Reoviridae family [1]. The virus contains a triple layered capsid, where the inner capsid layer of virus protein 2 (VP2) is surrounded by virus protein 6 (VP6) and forms an intermediate capsid layer. VP2 and VP6 layers are transcriptionally active. The outer capsid layer is composed of virus protein 7 (VP7) with spikes of virus protein 4 (VP4) forming the transcriptionally inactive mature RV virion [1]. VP4 and VP7 define the viral genotypes and are targets for neutralizing antibodies [2], [3], [4]. RV strains have been classified into 7 groups, A-H, based on antibody reactivity to the VP6 capsid protein [5], [6], [7]. Human RV strains are contained within groups A-C and H, with group A strains causing the majority of human illnesses globally [8]. Vaccination is the best means of preventing severe RV disease, and in 2009, the World Health Organization (WHO) recommended rotavirus vaccines for priority inclusion in national immunization programs worldwide. The Global Alliance for Vaccines and Immunization (GAVI) assists low-income countries to implement RV vaccination, however many non-GAVI countries with lower socioeconomics are unable to access vaccines at affordable costs, namely due to cold chain requirements for storage and delivery of RV vaccines [9], [10], [11], [12], [13]. This adds a burden to RV vaccination programs, particularly as vaccine titers are negatively affected by cold chain failures resulting in the need to administer greater volumes of vaccine [14]. There are 4 RV vaccines that have WHO prequalification and are available for procurement via GAVI, e.g. Rotarix, RotaTeq, Rotavac and Rotasil. Of these, Rotarix has been introduced into the largest number of countries while Rotavac and Rotasil are new products that countries are just now starting to consider. Rotarix is a monovalent, human, live-attenuated RV vaccine and prevents the replication of G1 and non G1 type RVs when administered as a 2-dose series in infants [15]. CDC-9 is a human strain of the natural reassortant with the VP3 gene derived from a G2P4 virus and the other 10 genes from a G1P8 virus, and the most common rotavirus genotype throughout the world [16]. This strain currently is under development for a new oral or an injectable vaccine against rotavirus infection in children. Rotavac is a monovalent human live-attenuated vaccine administered as a 3 dose series. Rotasil is the first RV vaccine with heat stable characteristics making it suitable for use in low-income countries where weaker infrastructure and frequent lack of electricity make refrigeration very difficult. The 116E RV vaccine strain is a reassortant strain of G9P11 containing one bovine rotavirus gene P [11] and 10 human rotavirus genes [17]. RV vaccine 116E was shown to have similar efficacy as Rotarix in developing countries [18]. These vaccines have had a major effect on RV morbidity and mortality [19], however the realization that RV vaccines are constrained because of supply and the need for cold chain capacity emphasizes the need for improved Vero cell vaccine cell substrates.

Vero cells are an optimal vaccine substrate for the production of many vaccines as this platform is accepted by Regulatory Authorities in >60 countries worldwide, and has been used for the production of licensed vaccines for >30 years, e.g., polio and rabies [20], [21]. Vero cells characterized using a broad range of tests to establish its suitability for vaccine production are a continuous cell line offering the ability for production using serum-free media and Good Manufacturing Practice (GMP) [22]. However, Vero cell lines typically have moderate vaccine yields. Live-attenuated RV vaccine preparation involves the use of RV seed lots to infect Vero cells producing predictable yields of virus (10^7^ virus particles/ml) to be used as an inoculum for bulk vaccine production [23], [24]. Given that a typical RV vaccine requires a delivery dose of 10^6^-10^7^ virus particles/ml [25], the efforts required for large-scale RV vaccine manufacturing are also great. Thus, a Vero cell substrate with enhanced RV replication or antigen expression could reduce the time, effort, and cost necessary to create enough vaccine viruses.

We previously published a dataset containing a genome-wide RNA interference (RNAi) screen that identified silencing events that enhanced RV replication, and evaluated several gene hits against multiple RV vaccine strains [26]. Vero cell cell lines were generated using a CRISPR-Cas9 platform where Sanger sequencing confirmed the gene knockout (KO). Unfortunately, the gene edited Vero cell lines tested did not substantially increase RV titer in large scale [27]. Thus, it remained possible that despite Sanger sequencing confirmation of gene knockout (KO) by CRISPR-Cas9 targeted gene editing there was still a wild type allele contributing to a heterozygous state. To improve on the development of KO Vero cells lines with increased RV vaccine capacity, we performed CRISPR-Cas9 gene editing to generate KO cells and confirmed KO by next-generation sequencing (NGS).

Building upon our previous findings using small interfering RNA (siRNA) libraries [28], [29], [30], a subset of genes was identified that increased RV replication in Vero cells that was validated by qRT-PCR [28]. Specifically, at two days post-siRNA transfection of African green monkey kidney cells (MA104), the cells were infected (MOI = 0.1) with RV3 (RV3-BB) [31], and at 48 h post-infection (hpi) the level of RV3 antigen was assessed using RV enzyme immunoassay (EIA). Knock down (KD) of 70 gene hits were examined for their ability to increase RV replication, i.e. ≥3 standard deviations (SDs) above the non-targeting control [26]. 10 of the 70 gene hits were re-screened using a fully-characterized WHO-certified Vero cell line, and found to increase RV replication (>3SD) at 48hpi by EIA and confirmed by qRT-PCR to silence >95% gene expression compared to non-targeting control siRNAs. Of the genes examined, KD of *NEU2*, *NAT9*, *COQ9*, *SVOPL*, *NDUFA9*, *COX9*, *EMX2*, *LRGUK*, *WDR62*, *RAD51AP1*, or *CDK6* genes resulted in a ≥2-fold increase in RV3 replication, and all KDs had a >95% KD of gene expression.

In the current study, the host genes restricting RV replication were selected for gene deletion using CRISPR-Cas9 [32]. Specifically, the leucine rich repeats and guanylate kinase domain containing gene (*LRGUK*) was targeted as the previous RNAi screen revealed a role for this gene in increasing vaccinia virus infection [33]. Similarly, WD repeat-containing protein (*WDR62*), identified to be involved in RNA splicing [34], was selected for gene editing, and the Empty Spiracles Homeobox 2 (*EMX2*) gene encodes a homeobox-containing transcription factor that may regulate mRNA transport or translation [35] but research on this gene in humans has focused on its expression in dorsal telencephalon, olfactory neuroepithelium, and urogenetial systems. Briefly, a guide RNA (gRNA) was used to target the specific genetic locus that was then cut by the Cas9 nuclease to generate double-strand breaks [36]. These cuts were endogenously repaired by non-homologous end joining (NHEJ) or by homology-directed repair (HDR) to create site-specific host gene KOs. The CRISPR-Cas9 plasmids used a double-nicking strategy to reduce off-target effects [37], and were transfected into Vero cells. GFP-positive transfected Vero cells were fluorescence activated cell sorting (FACS) sorted and the clones were evaluated using Illumina NGS to determine which clones contained the desired gene KOs, i.e. having a disrupted coding frame in the target gene were functionally tested to validate increased RV replication and antigen expression.

The Vero KO cell lines (substrates) were designated as ΔLRGUK Vero cells, ΔWR62 Vero cells, or ΔEMX2 Vero cells, and these cell lines and their clones were tested for increased RV replication and vaccine antigen expression compared to wild type Vero cells. The results showed that RV replication and antigen production was superior and several logs higher in ΔEMX2 cells compared to other cell substrates tested, while RV replication and antigen expression were appreciably higher in ΔWR62 Vero cells compared to ΔLRGUK Vero cells that were even higher than wild type Vero cells. The magnitude of RV replication and antigen expression was independent of virus strain differences in ΔEMX2 Vero cell substrates, but was dependent on strain differences in ΔWR62, ΔLRGUK and wild type (WT) Vero cells where CDC-9, Rotarix, 116E were propagated and the amount of RV antigen and virus replication were determined by a fluorescent focus assay [38].

## 2 Materials and Methods

### 2.1 Viruses

The RV strain (RV3-BB) used for the initial screen and validation studies is a naturally occurring human RV strain isolated from healthy neonates in Melbourne, Australia, and contains a G3 VP7 and a P[6] VP4 outer capsid [17], [39], [40]. The RV3, CDC-9, Rotarix, and 116E vaccine strains were propagated in Vero cells using an MOI = 0.5. CDC-9 is culture-adapted and encodes a VP3 gene related to that of the DS-1 strain (ATCC VR2550) and other 10 genes with identity to G1P [8] RV strains of RV [16]. Rotarix is a G1P [8] rotavirus strain isolated from a child with gastroenteritis and adapted from a vaccine vial at the CDC. The 116E strain is a naturally occurring reassortant strain G9P [11] containing one bovine rotavirus gene (P [11]) and 10 human rotavirus genes [41].

### 2.2 Cell lines

Vero cells from a low-passaged African green monkey kidney cell line were used [21]. The Vero cell line was obtained from ATCC, CCL81.4, lot #738812 at passage 123. ΔWDR62, ΔLRGUK, ΔEMX2 Vero cells and WT Vero cells were all grown in high glucose DMEM (GIBCO) + 5% fetal bovine sera (FBS) (Hyclone) (DMEM-5%). A master cell stock was created for low-passaged WT and gene-edited KO Vero cell lines and stored in liquid N_2_ vapor.

### 2.3 RV infection of cells

RV was activated with 50ug/ml trypsin (ThermoFisher), diluted in the DMEM, and heated for 1 h in a 37 °C water bath. Post-activation, the culture media was removed from plates containing Vero cells and washed 2 × with PBS to remove residual FBS. RV was added (0.1 ml) to each well of plated cells. Infected plates were incubated for 1 h at 37 °C, 5% CO**_2_**. Post-infection, supernatants were transferred to fresh cells for 16 h and harvested for evaluation by EIA. Following the incubation, the cells were fixed with 4% formalin for 20–30 min and FFA or fluorescent focus unit (FFU) quantification was performed using a Cellomics ArrayScan VTI HCS Reader (Thermo Fisher). RV was propagated in Vero cells and expanded in WT or KO Vero cell substrates, e.g. ΔWDR62, ΔLRGUK or ΔEMX2 Vero cells. Rotarix, CDC-9 and 116E were examined in the studies.

### 2.4 siRNA examination

A sub-library of ON-TARGETplus siRNA (SMARTpools; GE Healthcare) was individually transfected into WT Vero cells as described [26]. Each transfection experiment included a non-targeting control (NTC) siRNA, a siRNA targeting RV3 (positive control), and siTOX (GE Healthcare) served as a transfection control where transfection resulted in cell death. All siRNAs were transfected to a final concentration of 50 nM.

### 2.5 CRISPR-Cas9

Single guide RNA **(**sgRNAs) were designed to identify guide sequences and minimize identical genomic matches or near matches to reduce off-target effects. The guide sequences consisted of a protospacer sequence upstream of a protospacer adjacent motif (PAM) recognition site. For creating a gene KO, two sgRNAs located within exon were used to produce clones with a loss of function, i.e., a frame-shift mutation.

#### 2.5.1 Design of deletion screening primers

A set of primers internal to the sequence to be deleted and another set of primers upstream and downstream of the sgRNA cleavage sites were used. A pair of forward and reverse primers flanking each sgRNA target site were used to amplify the sgRNA target site to characterize the non-deleted allele in monoallelic deletion clones.

#### 2.5.2 CRISPR cloning

Oligomers (oligos) were annealed and phosphorylated using standard procedures, i.e. using a thermocycler at 37 °C for 30 min; 95 °C for 5 min, and then ramping down to 25 °C at 5 °C/min. The annealed oligos were ligated using a Golden Gate assembly cloning strategy as previously described [42], [43]. Samples were run in a thermocycler using the following parameters: Cycles 1–20 (37 °C for 5 min, 20 °C for 5 min); Cycle 21 (80 °C for 20 min). *E. coli* cells were transformed, plated with 100 μg/ml ampicillin, and incubated overnight at 37 °C. Colonies were picked and inoculated into mini-prep cultures and sequence-verified prior to inoculation into a maxi-prep culture. Maxi-preps were done for each CRISPR/Cas9 construct.

#### 2.5.3 Transfecting CRISPRs into Vero cells

Vero cells were transfected using Lipofectamine LTX (Life Technologies). Cells were seeded at 80% confluence into 6-well plates 16 h prior to transfection. Lipofectamine LTX (6.25 µl) was diluted into 100 µl OPTI-MEM. CRISPR DNA (3.75 µg) was added to 100 µl of OPTI-MEM. The transfection reagent was added to the DNA and allowed to incubate at room temperature (RT) for 30 min before adding to the cells. The medium was changed 24 h after transfection, and GFP was detected 48 h post-transfection. The cells were then sorted based on GFP fluorescence in which top ~5% of GFP-positive cells were seeded individually into 96-well round-bottom plates.

#### 2.5.4 Screening for CRISPR-Cas9 deletions

Genomic DNA (gDNA) was isolated from sorted cells. PCR was used to validate primers and verify the presence of the intended genomic deletion. Samples were run in a thermocycler and separated on a 2% agarose gel to screen for the presence/absence of gene-deletion bands. Vero cells (100 µl) were plated into two separate 96-well flat-bottom plates. One plate was incubated at 37 °C and the other plate was used to screen each clone for deletions. gDNA was extracted from the clones, and each clone was screened using the same optimized PCR primers and reaction conditions. Clones with the desired deletion were identified and expanded.

### 2.6 Eia

WT and KO Vero cells (ΔWDR62, ΔLRGUK, ΔEMX2) were cultured in 96-, or 24-well plates for assays and infected with RV strains Rotarix, CDC-9, or 116E for 3 days or 5 days at a MOI of 0.1 or 0.2, respectively. Following incubation, supernatants were evaluated by EIA. Briefly, cell culture supernatants were collected (50 µl) and used to coat a 96-well EIA (ThermoFisher) overnight at 4 °C on a rocker. Following incubation, plates were washed 3 × with KPL wash buffer (Thermo Fisher), and blocked with blocking solution (5% nonfat dry milk in KPL buffer) for 1 h at RT. Blocking buffer was removed and 50 µl of a 1:1000 dilution of primary rabbit anti-RV polyclonal sera (Rab anti-SA11) in blocking buffer was added and incubated on a rocker for 1 h at RT. Plates were washed 3 × with KPL and 50 µl of HRP-conjugated goat/anti-rabbit secondary antibody (1:800) in blocking solution was added and incubated on a rocker for 1 h at RT. Plates were washed 3 × with KPL. TMB substrate (100 µl) (Sigma) was added to each well and incubated for 15 min in the dark at RT. TMB reaction was stopped with 100 µl of stop solution. Plates were read at 450 nm using an EPOCH plate reader (BioTek).

### 2.7 Ffa

96-wells plates were used for the FFA assays. The inoculum was removed and cells fixed with 4% formalin and the fixed FFA plates were washed 2 × with PBS and blocked for 1 h at RT with blocking solution. Blocking solution was discarded and the primary polyclonal rabbit anti-RV antibody, diluted 1:1000 in blocking solution, was added to each well (50 µl), and incubated for 1 h at RT. The primary antibody solution was removed, and the plates were washed 3 × with KPL wash buffer, followed by the addition of a goat anti-rabbit Alexa 488 (Thermo Fisher) at 1:500 in blocking solution for 1 h at RT. The secondary antibody was removed, and the plates were then washed 3 × with KPL wash buffer. Plates were stained with DAPI (1:10,000 in PBS) for 20 min at room temperature. Plates were washed 3 × with KPL wash buffer. PBS (100 µl) was added to each well. Plates were imaged using Cellomics ArrayScan VTI HCS Reader (20 × magnification). Titer (FFU/ml) was calculated by counting the number of fluorescent foci in the highest and second highest dilutions, calculating FFU/ml and calculating the average.

### 2.8 Statistical analysis

Statistical analysis was performed using a one-way analysis of variance with Tukey’s multiple-comparisons test at the 95% confidence level. All data are presented as mean ± SE. *p*-values < 0.05 were considered statistically significant.

## 3 Results

The Vero WT and KO cell lines were maintained in the exponential phase of replication, i.e. sub-cultured regularly before they entered the stationary growth phase or before the cell monolayer became 100% confluent. The WT Vero cell cultures divided at a uniform rate and were evaluated against the generational time of ΔWDR62, ΔLRGUK and ΔEMX2 cells by determining the mean generational time but no significant differences were observed following culture in a 96-well or 24-well tissue culture plate.

**Fig. 1:**
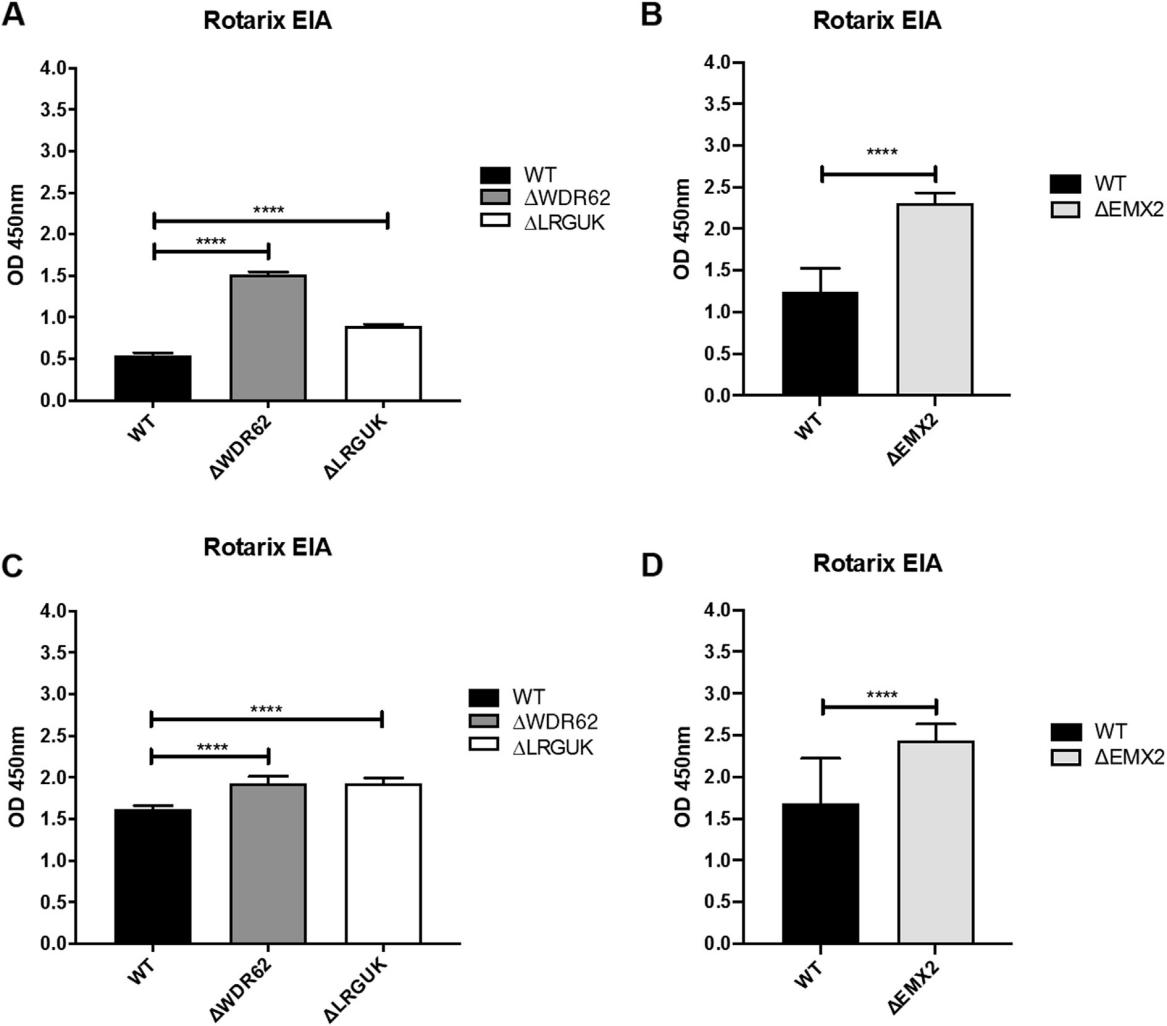
Enzyme immunoassay (EIA) of Rotarix. WT, ΔWDR62, ΔLRGUK, or ΔEMX2 cells were infected with Rotarix MOI 0.1 in 96-well format for 3 days (A,B) or MOI 0.2 in 24-well format for 5 days (C,D) Supernatants were collected and assayed by EIA for RV antigen using an anti-RV rabbit polyclonal serum and goat/anti-rabbit HRP conjugated IgG secondary. Following TMB substrate reaction/stop buffer plates were read at 450 nm using an EPOCH plate reader. Data represent ± SEM from six independent replicates. Differences in absorbance were compared using one-way ANOVA ****p < 0.0001.

**Fig. 2:**
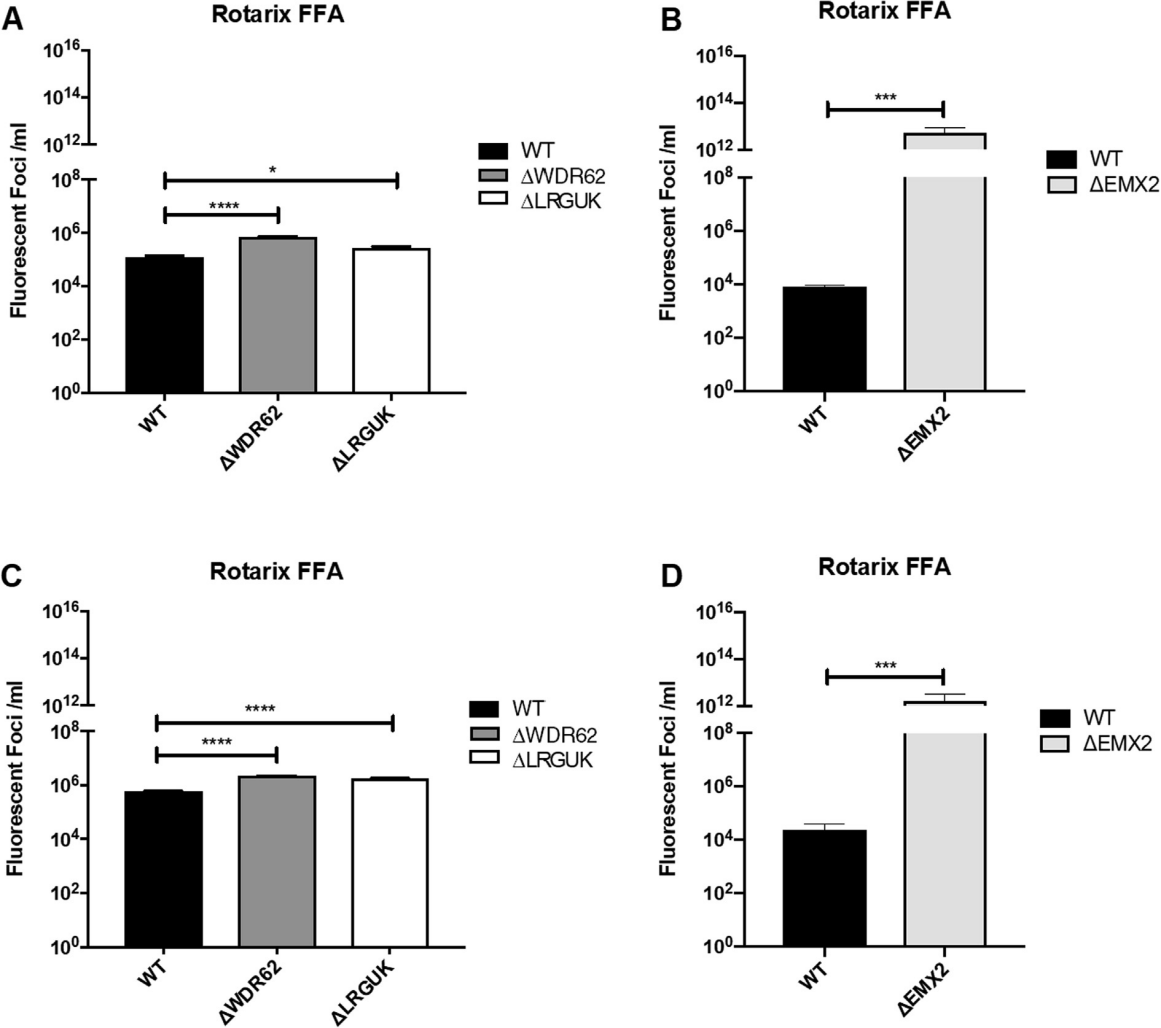
Fluorescent focus assay (FFA) showing Rotarix replication. WT, ΔWDR62, ΔLRGUK, or ΔEMX2 cells were infected with Rotarix MOI 0.2 in 96-well format for 3 days (A,B) or in 24-well format for 5 days (C,D) followed by transfer of supernatants to fresh cells for 16 h. Cells were washed, fixed with 4% formalin, and stained for RV antigen using an anti-RV rabbit polyclonal serum primary and goat anti-rabbit Alexa 488 fluorescent secondary. Cells (n > 20,000) were imaged on Arrayscan VTI HCS Reader. Titers were calculated by counting fluorescent foci in the highest and second highest infected sample dilutions. These titers were then averaged for each sample. Data represent ± SEM from six independent replicates. Differences in fluorescent foci were compared using one-way ANOVA *p < 0.01; ****p < 0.0001.

**Fig. 3:**
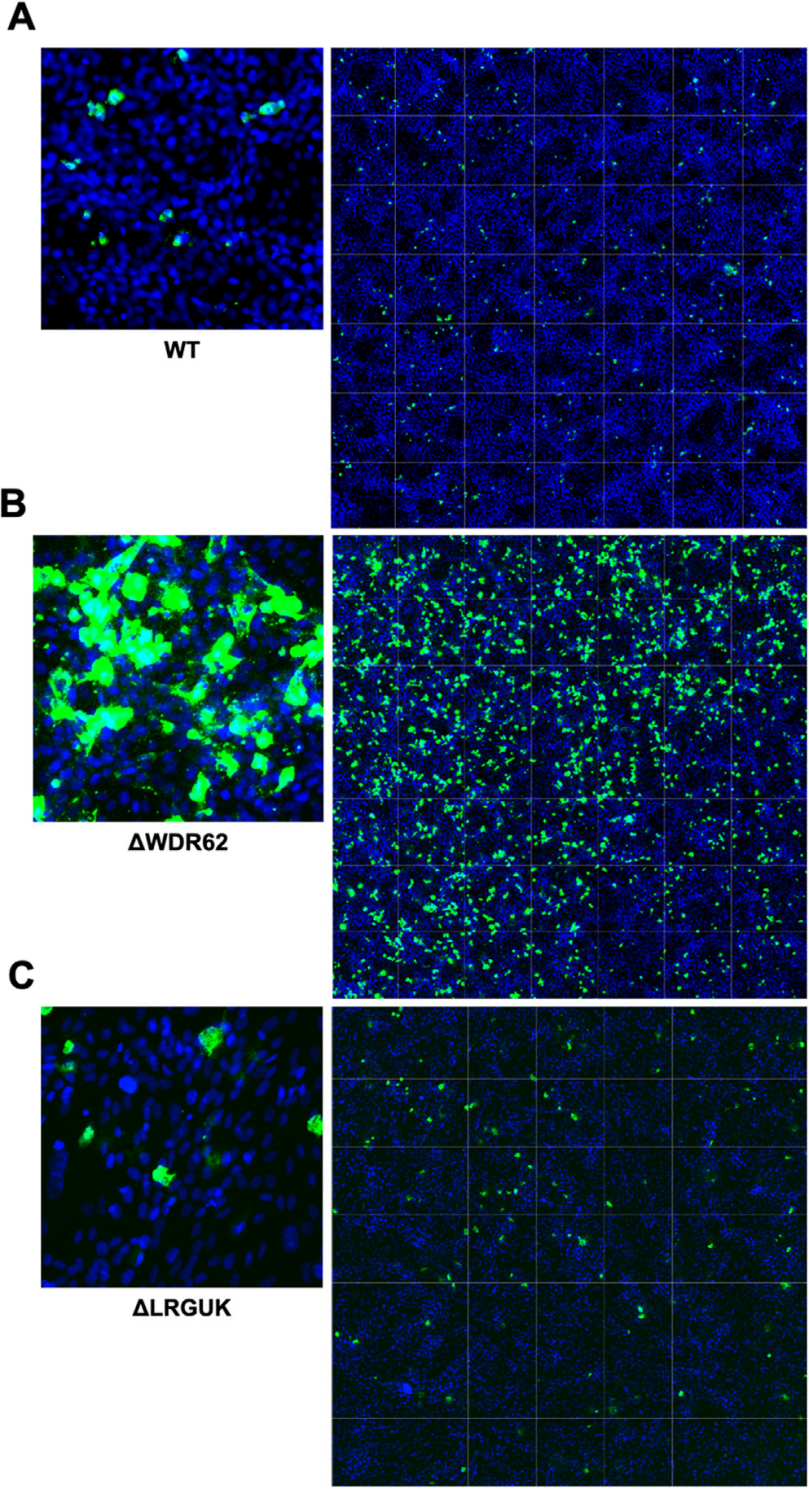

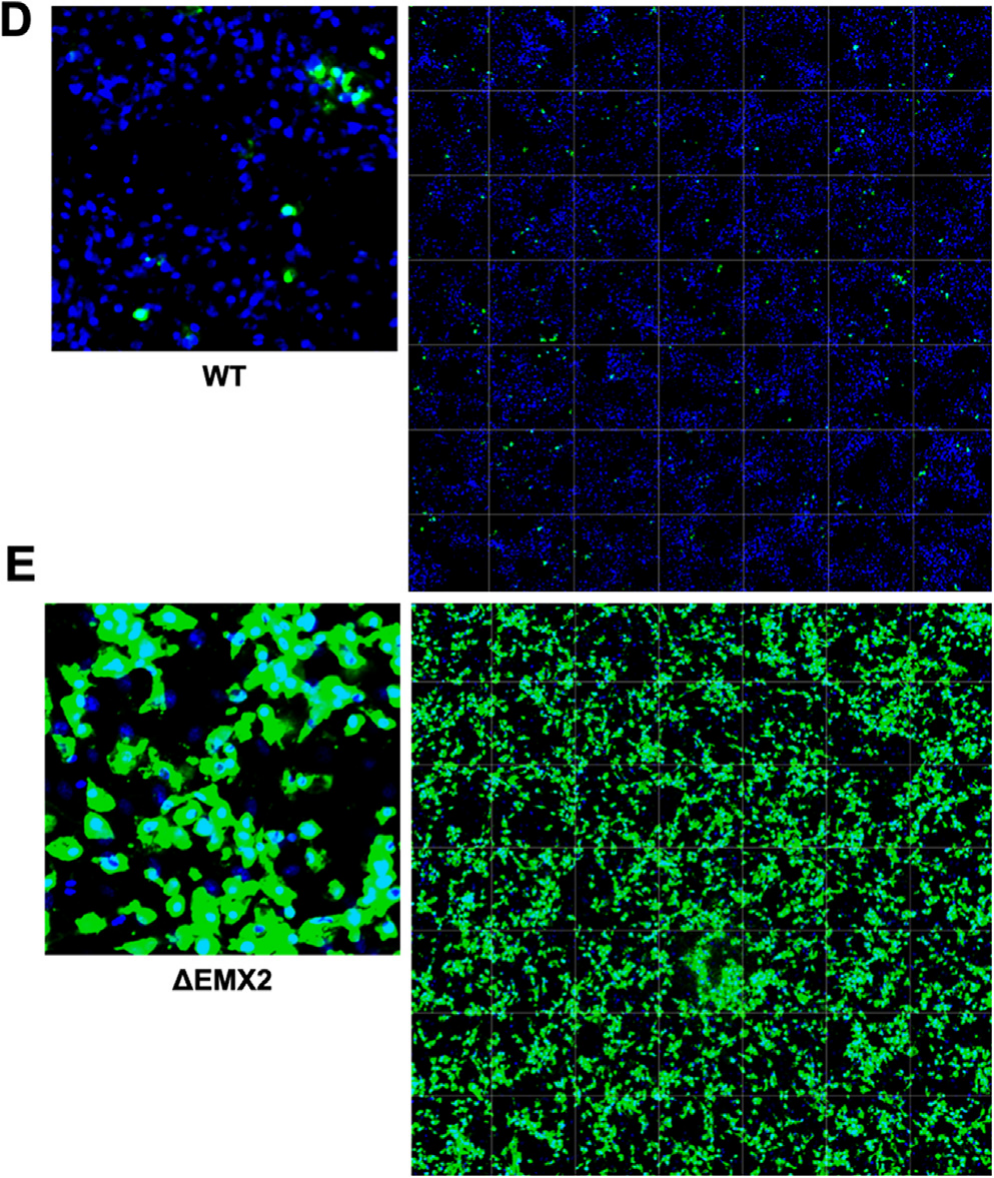
Imaging of Rotarix. WT (A,D), ΔWDR62 (B), ΔLRGUK (C), or ΔEMX2 (E) cells were infected with Rotarix MOI 0.1 in 96-well format for 3 days followed by transfer of supernatants to fresh cells for 16 h. Cells were washed, fixed with 4% formalin and stained for RV antigen using an anti-RV rabbit polyclonal serum primary and goat anti-rabbit Alexa 488 fluorescent secondary. Cells (n > 20,000) were imaged on Arrayscan VTI HCS Reader at 20 × magnification. Shown is an enlarged representative field (left) and an image containing a representative population of cells (>10,000) (right).

**Fig. 4:**
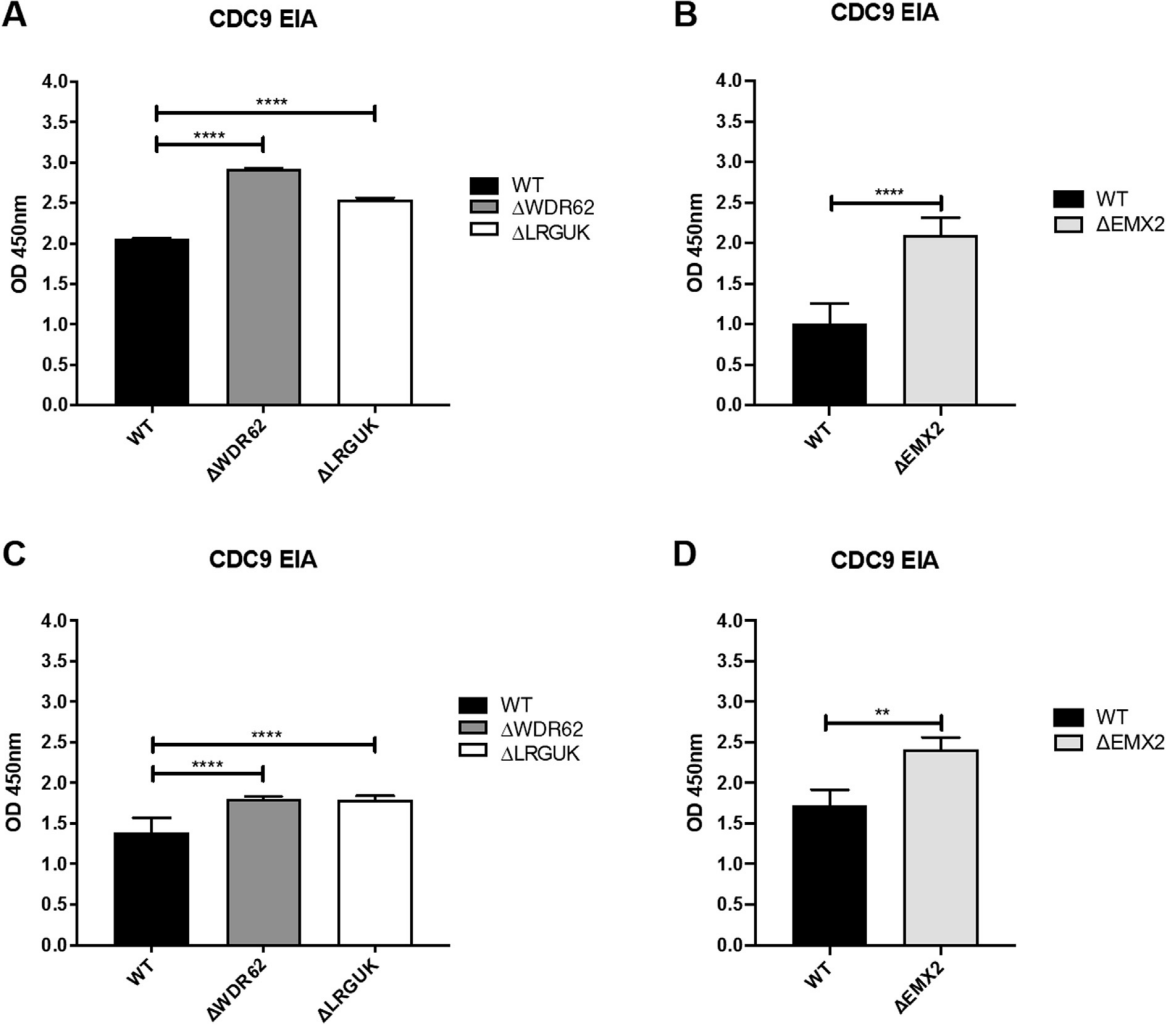
Enzyme immunoassay (EIA) of CDC-9. WT, ΔWDR62, ΔLRGUK, or ΔEMX2 cells were infected with CDC-9 MOI 0.1 in 96-well format for 3 days (A,B) or MOI 0.2 in 24-well format for 5 days (C,D) Supernatants were collected and assayed by EIA for RV antigen using an anti-RV rabbit polyclonal serum and goat/anti-rabbit HRP conjugated IgG secondary. Following TMB substrate reaction/stop buffer plates were read at 450 nm using an EPOCH plate reader. Data represent ± SEM from six independent replicates. Differences in absorbance were compared using one-way ANOVA ****p < 0.0001.

**Fig. 5:**
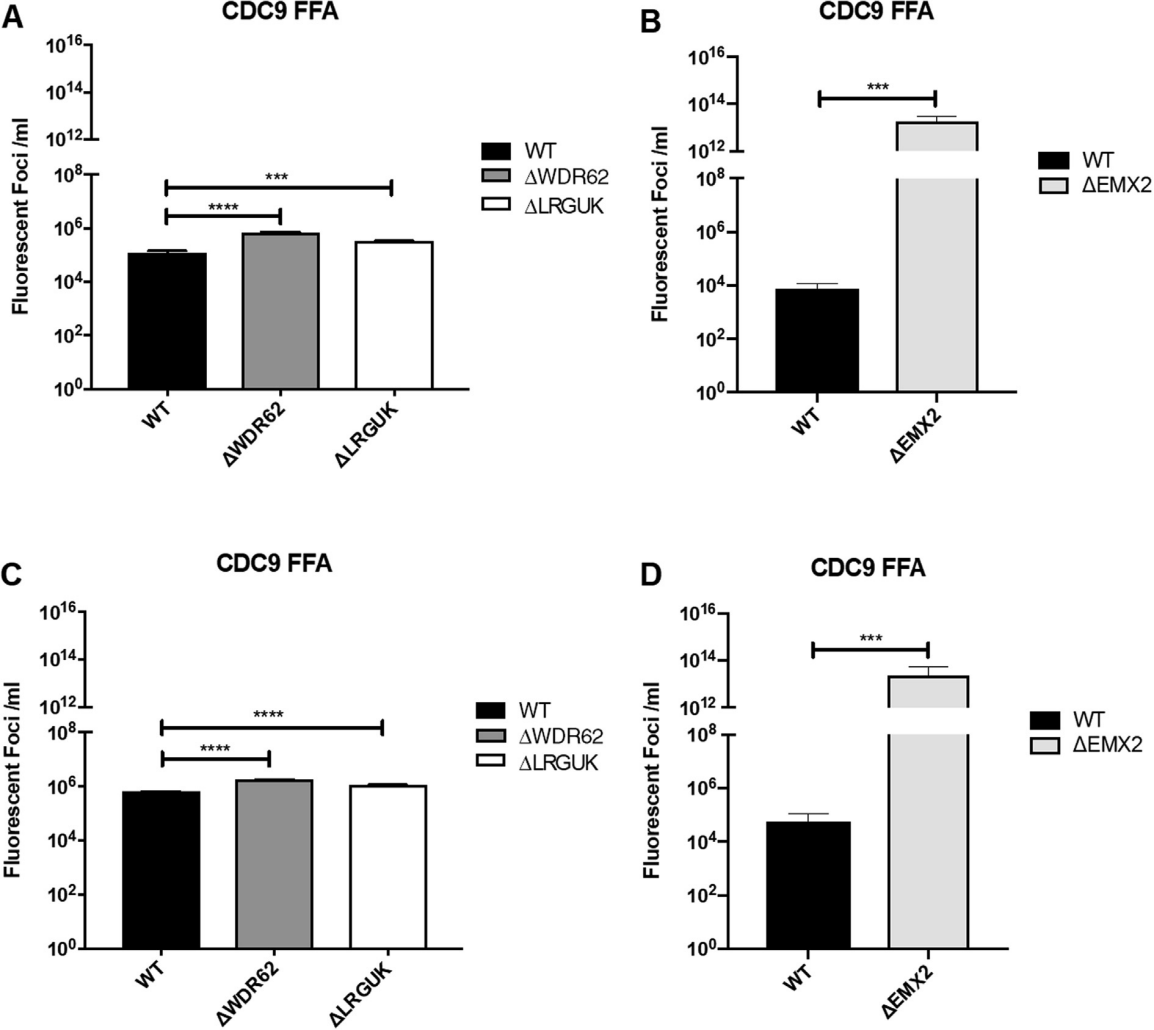
Fluorescent focus assay (FFA) showing CDC-9 replication. WT, ΔWDR62, ΔLRGUK, or ΔEMX2 cells were infected with CDC-9 MOI 0.2 in 96-well format for 3 days (A,B) or in 24-well format for 5 days (C,D) followed by transfer of supernatants to fresh cells for 16 h. Cells were washed, fixed with 4% formalin and stained for RV antigen using an anti-RV rabbit polyclonal serum primary and goat anti-rabbit Alexa 488 fluorescent secondary. Cells (n > 20,000) were imaged on Arrayscan VTI HCS Reader. Titers were calculated by counting fluorescent foci in the highest and second highest infected sample dilutions. These titers were averaged for each sample. Data represent ± SEM from six independent replicates. Differences in fluorescent foci were compared using one-way ANOVA *p < 0.01; ****p < 0.0001.

**Fig. 6:**
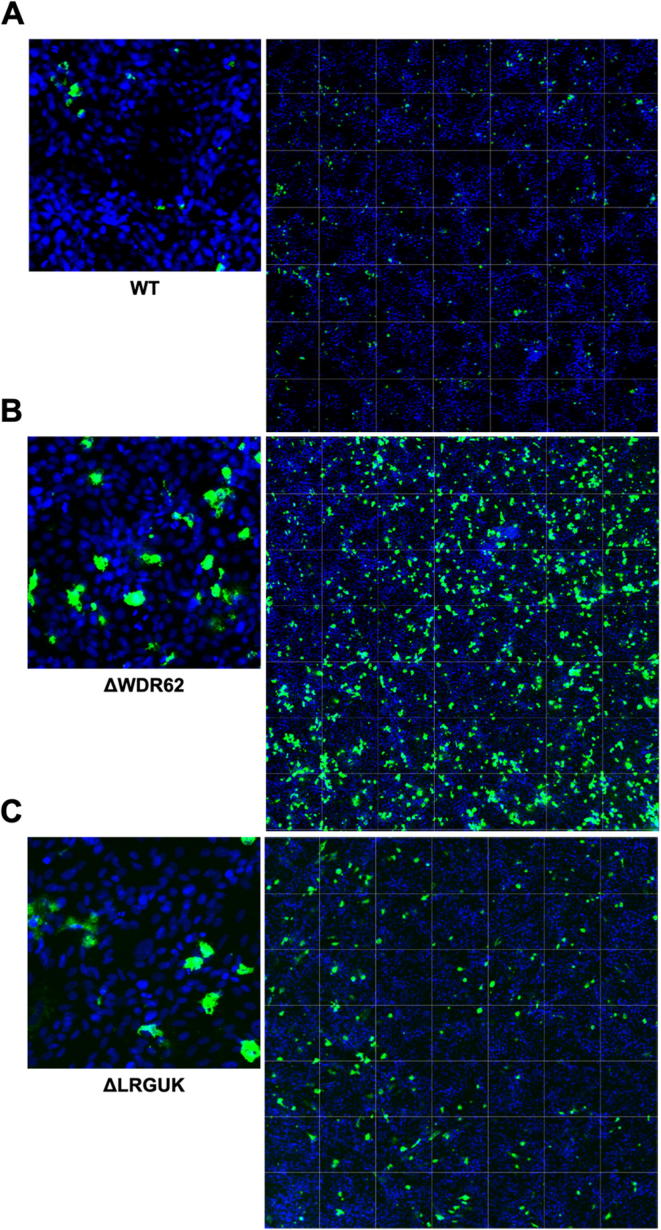

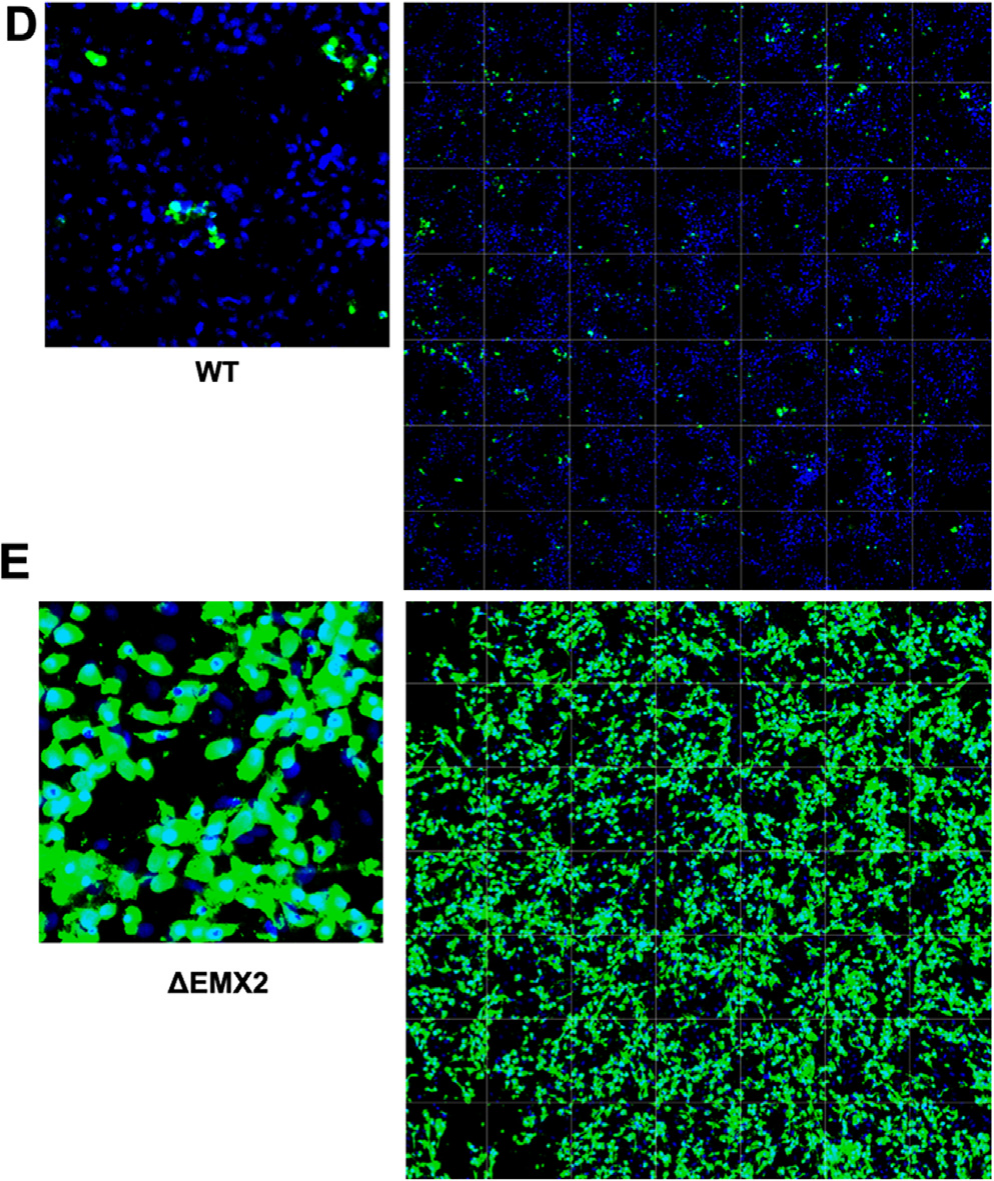
Imaging of CDC-9. WT (A), ΔWDR62 (B), ΔLRGUK (C), or ΔEMX2 (D) cells were infected with CDC-9 MOI 0.1 in 96-well format for 3 days followed by transfer of supernatants to fresh cells for 16 h. Cells were washed, fixed with 4% formalin, and stained for RV antigen using an anti-RV rabbit polyclonal serum primary and goat anti-rabbit Alexa 488 fluorescent secondary. Cells (n > 20,000) were imaged on Arrayscan VTI HCS Reader at 20 × magnification. Shown is an enlarged representative field (left) and an image containing a representative population of cells (>10,000) (right).

**Fig. 7:**
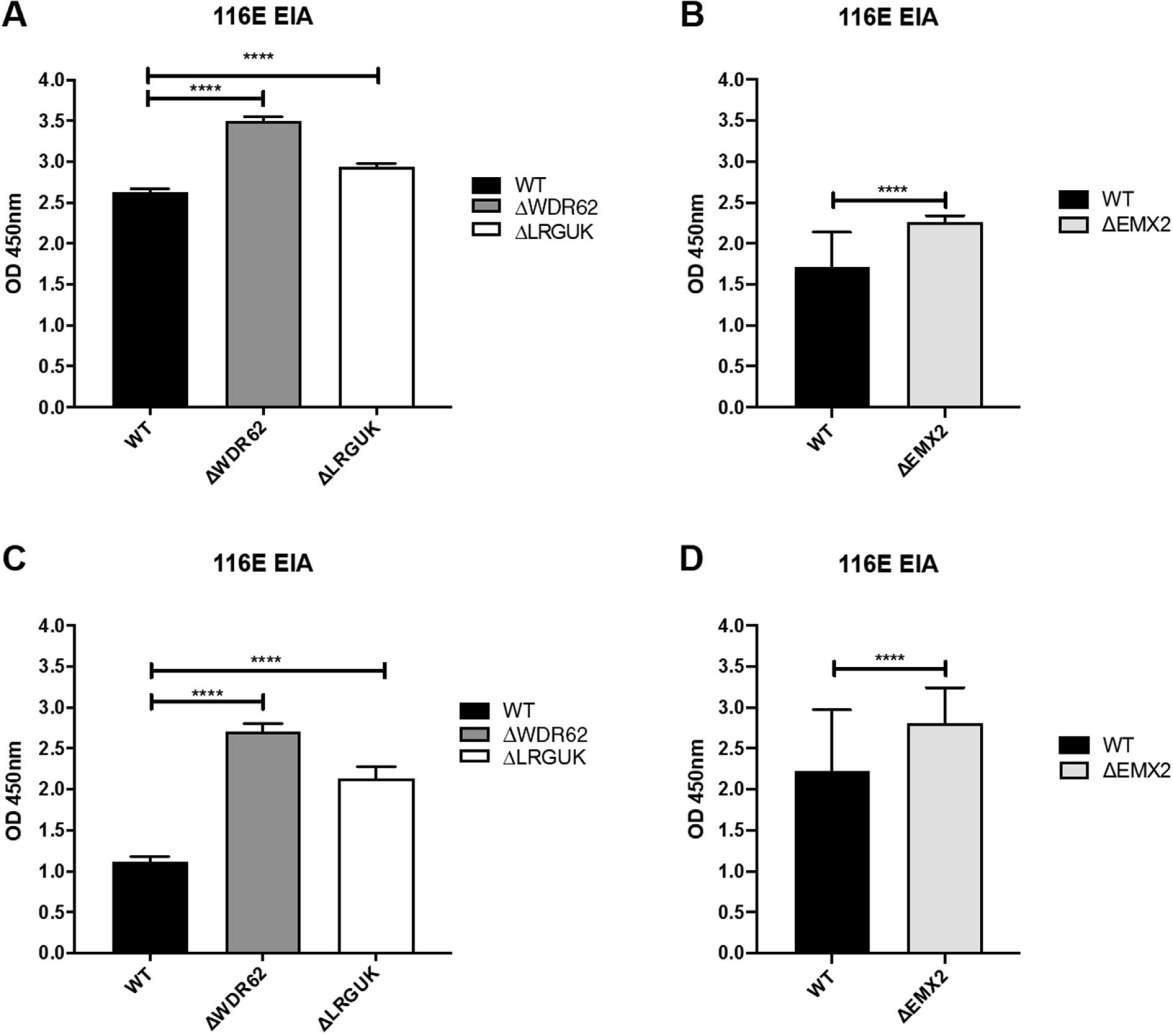
Enzyme immunoassay (EIA) of 116E. WT, ΔWDR62, ΔLRGUK, or ΔEMX2 cells were infected with 116E MOI 0.1 in 96-well format for 3 days (A,B) or MOI 0.2 in 24-well format for 5 days (C,D) Supernatants were collected and assayed by EIA for RV antigen using an anti-RV rabbit polyclonal serum and goat/anti-rabbit HRP conjugated IgG secondary. Following TMB substrate reaction/stop buffer plates were read at 450 nm using an EPOCH plate reader. Data represent ± SEM from six independent replicates. Differences in absorbance were compared using one-way ANOVA ****p < 0.0001.

**Fig. 8:**
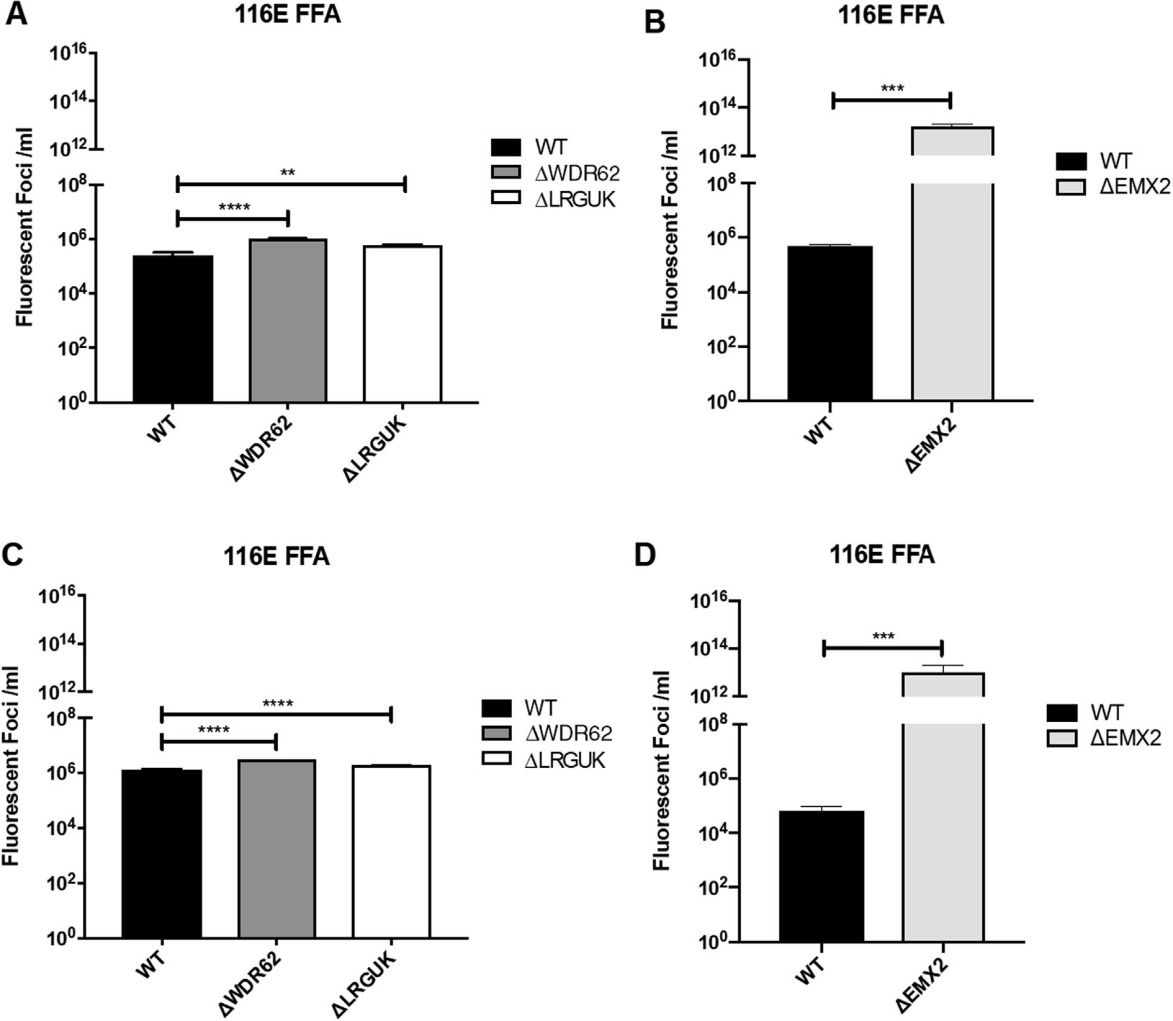
Fluorescent focus assay (FFA) showing 116E replication. WT, ΔWDR62, ΔLRGUK, or ΔEMX2 cells were infected with 116E MOI 0.2 in 96-well format for 3 days (A,B) or in 24-well format for 5 days (C,D) followed by transfer of supernatants to fresh cells for 16 h. Cells were washed, fixed with 4% formalin, and stained for RV antigen using an anti-RV rabbit polyclonal serum primary and goat anti-rabbit Alexa 488 fluorescent secondary. Cells (n > 20,000) were imaged on Arrayscan VTI HCS Reader. Titers were calculated by counting fluorescent foci in the highest and second highest infected sample dilutions. These titers were averaged for each sample. Data represent ± SEM from six independent replicates. Differences in fluorescent foci were compared using one-way ANOVA *p < 0.01; ****p < 0.0001.

**Fig. 9:**
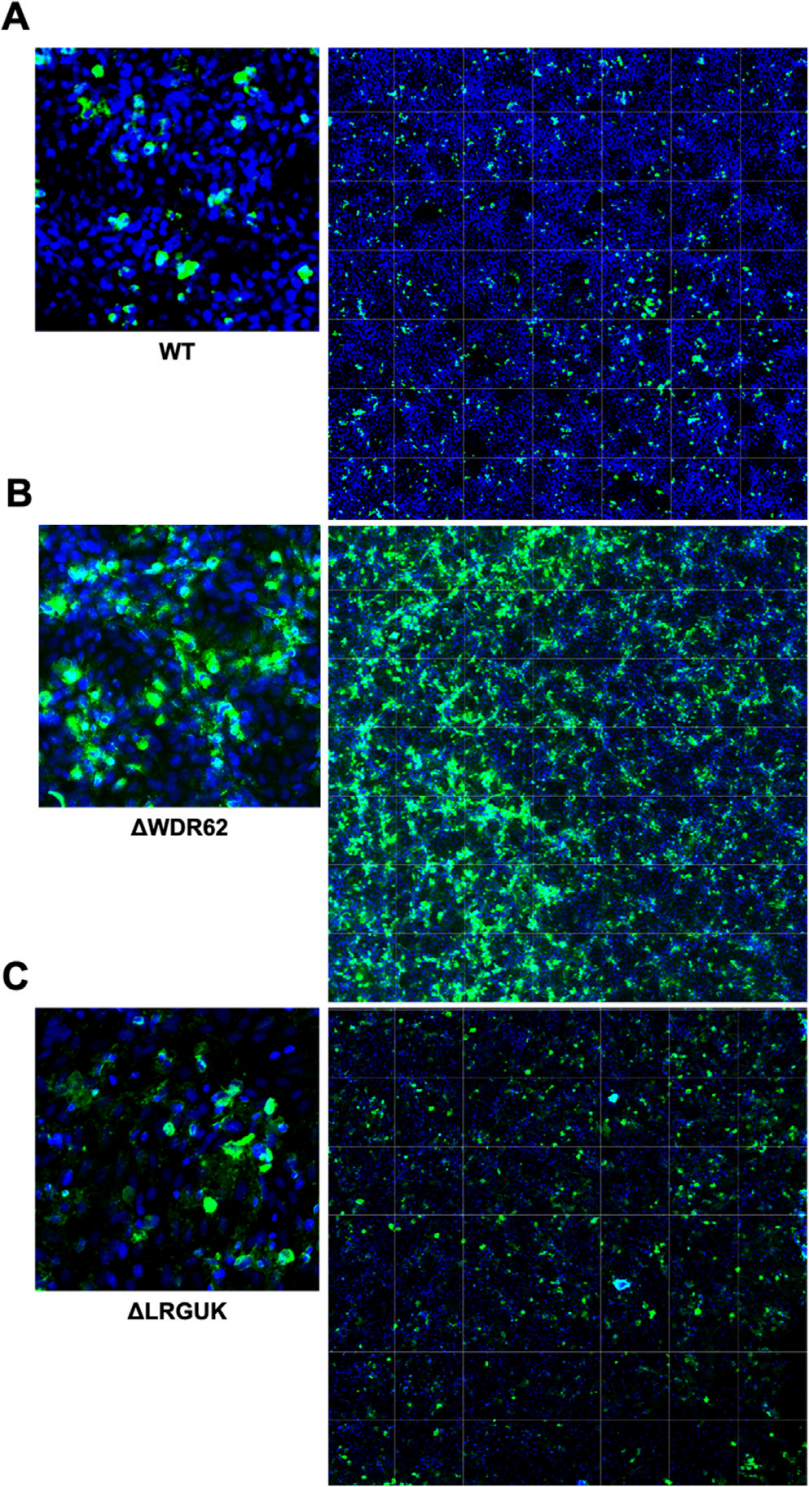

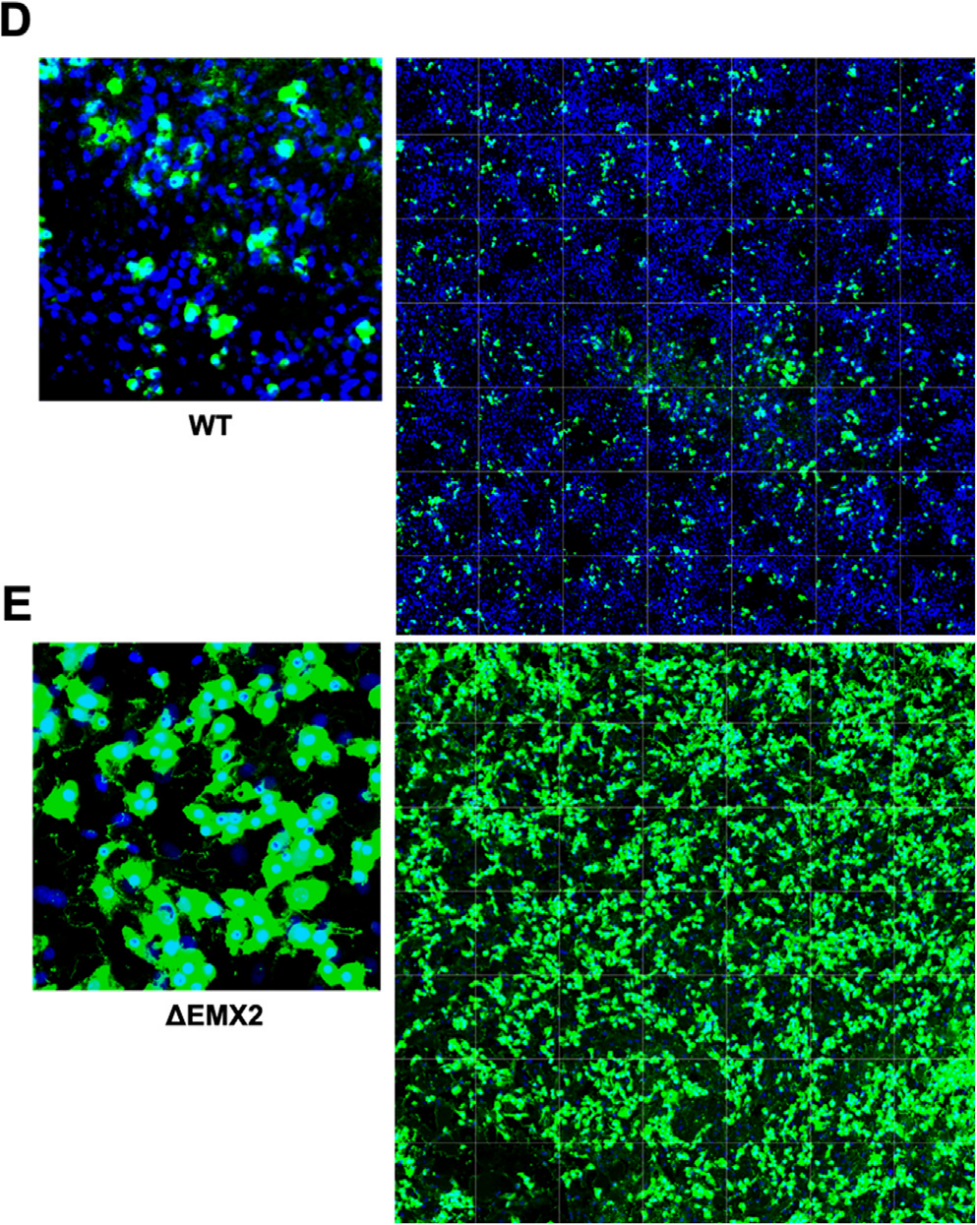
Imaging of 116E. WT (A), ΔWDR62 (B), ΔLRGUK (C), or ΔEMX2 (D) cells were infected with 116E MOI 0.1 in 96-well format for 3 days followed by transfer of supernatants to fresh cells for 16 h. Cells were washed, fixed with 4% formalin, and stained for RV antigen using an anti-RV rabbit polyclonal serum primary and goat anti-rabbit Alexa 488 fluorescent secondary. Cells (n > 20,000) were imaged on Arrayscan VTI HCS Reader at 20 × magnification. Shown is an enlarged representative field (left) and an image containing a representative population of cells (>10,000) (right).

The WT and KO Vero cell lines were evaluated for their permissiveness to RV replication by EIA and FFA assays WT and KO cells were plated either in microplate format (96-well) or larger format (24-well) and infected with Rotarix (Fig. 1, Fig. 2, Fig. 3), CDC-9 (Fig. 3, Fig. 4, Fig. 5, Fig. 6), or 116E (Fig. 7, Fig. 8, Fig. 9). Antigen levels were determined by EIA (Fig. 1A–D, 4A–D, and 7A–D), viral titer (FFU/ml) (Fig. 2A–D, Fig. 5A–D, Fig. 8A–D), and by imaging and FFA (Fig. 3A–E, Fig. 6A–E, Fig. 9A–E). Replication of Rotarix (Fig. 1, Fig. 2, Fig. 3), CDC-9 (Fig. 4, Fig. 5, Fig. 6) and 116E (Fig. 7, Fig. 8, Fig. 9) was considerably increased in ΔWDR62 and ΔLRGUK but dramatically increased in ΔEMX2 cell substrates compared to WT Vero cells when evaluated by EIA, imaging, or FFA (Fig. 3D,E, Fig. 6D,E and Fig. 9D,E). The results showed that the magnitude of RV replication and antigen expression was independent of the RV vaccine candidates when propagated in ΔEMX2 cell substrates, but was dependent on the RV vaccine strain tested in KO or WT Vero cell substrates. Days 3 and 5 pi were assessed, and by day 5 pi the RV-infected EMX2-deleted Vero cells were obliterated compared to the other Vero cells. Importantly, the results show that ΔEMX2 Vero cell substrates, and to a lesser degree ΔWDR62 or ΔLRGUK Vero cells substrates, can increase RV vaccine antigen and RV titers offering the ability to affordably propagate RV vaccine candidates. The KO cell lines were sequenced by NGS (Hudson Alpha Institute, Huntsville, AL), and RV replication determined by EIA and fluorescent focus assays (FFA) as previously described [44], [45]. RV3 was used in the screen and validation studies [26], thus comparisons are made to the EIA antigen and FFA replication levels for CDC-9, Rotarix and 116E. (See Fig. 1, Fig. 2, Fig. 3, Fig. 4, Fig. 5, Fig. 6, Fig. 7, Fig. 8, Fig. 9)

## 4 Discussion

Vaccine manufacture requires producing large quantities of vaccine preparation in batches that are readily available and amenable for use in cell culture or other systems [46], [47], [48]. Difficulties associated with other vaccine substrates and concerns about adventitious viruses that could compromise the vaccine production provide rationale for using well-characterized Vero cell vaccine platforms [49]. Vero cells have proven to be a safe production platform for developing human vaccines [46], [48], [50], [51], [52], however Vero cells suffer from a relatively reduced virus yield compared to other vaccine platforms. To overcome these issues, we pursued the creation of an improved RV vaccine Vero cell substrate. This was attained as previously described where we (1) identified key host genes that affect RV replication, (2) identified a subset of host genes whose KD increased RV replication, (3) used siRNAs to target and validate the host genes, (4) silenced the genes of interest by RNAi and subsequently infected the KD cells with RV, and (5) determined the level of RV production at 3–5 days pi compared to WT Vero cells. Using fully-characterized Vero cells, the host genes identified were edited using CRISPR-Cas9. In this study, we examined RV replication and antigen expression in several KO Vero cell lines and showed that the magnitude was independent of the RV vaccine candidates when propagated in ΔEMX2 cell substrates, but was dependent on the RV vaccine strain tested in KO or WT Vero cell substrates. Importantly, the results show that ΔEMX2 Vero cell substrates, and to a lesser degree ΔWDR62 or ΔLRGUK Vero cells substrates, can increase RV vaccine antigen and infectious RV titers offering the ability to reduce the costs to propagate RV vaccines. As different clones of Vero cell may produce different yields of virus whether gene edited or not [53], [54], [21], it is possible that the WDR62 and LRGUK gene deleted Vero cell lines may be in this category where the differences in yield observed are not necessarily due to gene deletion. However, the RV vaccine candidates grew to ~5–7 logs higher virus titer over the same time-period in ΔEMX2 cell substrates compared to WT Vero cells, and ~2–3 logs higher titer in ΔWDR62 or ΔLRGUK Vero cell substrates. Importantly, there were no detectable antigenic changes in antibody reactivity by EIA or FFA assays of input or recovered RV yields.

The development of improved Vero vaccine cell line substrates offers a solution to overcome the cost and vaccine production hurdles. It will be necessary to determine if the KO cell substrates can yield rotavirus vaccines at large-scale allowing for a reduction of costs by minimizing cold storage, packaging, and shipping requirements. It is plausible that enhanced Vero cells vaccine substrates would meet the need for increased volume and lowered production costs that linked to prerequisites needed for smaller single-use bioreactors.

## Declaration of Competing Interest

The authors declare the following financial interests/personal relationships which may be considered as potential competing interests: [The authors report no relationships or activities that could appear to have influenced the submitted work. CK has a patent on the RV3 rotavirus vaccine. The findings and conclusions in this report are those of the authors and do not necessarily represent the official positons of Centers for Disease Control and Prevention]
